# Supplementary material for: Changes in infant non-nutritive sucking throughout a suck sample at 3-months of age
Source: PLoS One. 2020 Jul 9;15(7):e0235741. doi: 10.1371/journal.pone.0235741 (PMC7347146; doi:10.1371/journal.pone.0235741)
Supplement: Attachment — Submitted filename: Response to Reviewers_TC.docx [file pone.0235741.s002.docx]

Thank you to the associate editor and the reviewers for the opportunity to improve this manuscript. Please see the below responses to the reviewers’ comments. We feel that your insights greatly improved our manuscript.

4. Have the authors made all data underlying the findings in their manuscript fully available?

The [PLOS Data policy](https://nam12.safelinks.protection.outlook.com/?url=http%253A%252F%252Fwww.plosone.org%252Fstatic%252Fpolicies.action%2523sharing&data=02%257C01%257Ca.martens%2540northeastern.edu%257C917c114effde432b732008d80c62d1de%257Ca8eec281aaa34daeac9b9a398b9215e7%257C0%257C0%257C637272965880581717&sdata=OMrqoG8Dod03jHSyBUpM5AZU%252BRDLjP22R5yfL3Pr4uQ%253D&reserved=0) requires authors to make all data underlying the findings described in their manuscript fully available without restriction, with rare exception (please refer to the Data Availability Statement in the manuscript PDF file). The data should be provided as part of the manuscript or its supporting information, or deposited to a public repository. For example, in addition to summary statistics, the data points behind means, medians and variance measures should be available. If there are restrictions on publicly sharing data—e.g. participant privacy or use of data from a third party—those must be specified.

Reviewer #1: No

Response: The authors confirm that some access restrictions apply to the data underlying the findings. Data are confidential because the study uses personal data from minors and a clinical population. Data are available to researchers who qualify for access to confidential data. Requests can be made by contacting:

Northeastern University, 360 Huntington Ave, 132

Forsyth Building, Phone: 617-373- 4670

Email: m.hines@northeastern.edu.

Reviewer #1: The authors addressed most of my major concerns. In particular, they added a sensitivity analysis to assess the generalizability of the results given my concern about the variability in the number of bursts per infant. The authors say that the sensitivity analysis "confirmed overlapping breakpoint confidence intervals for each NNS outcome between the subset datasets and the entire cohort". Yet, it is not clear how this analysis was performed (statistical methods) neither the quantitative results are reported.
The authors should add some more quantitative details about such analysis. The description should be added to the methods and detailed results should be reported in the text or in a table.
Response: Thank you for the comment. The sensitivity analysis has been further qualified along with an addition of a table to further clarify the results (Table 3).

Regarding my previous comment: "Authors should include a table reporting the results (outcome measure, number of bursts, duration of data) separated by main groups (e.g. by sex, age, first time users, and so on..). Further statistics on the outcome measures should be performed, to report any possible difference between groups (first time users vs. not; male vs female)."

Authors added Table 1 to report main characteristics of the cohort and NNS burst number in F and M.
The other outcome measures should be also added. The authors state that no difference was found in all descriptors; however, no p-values are reported and nowhere in the manuscript they described which test was performed to test this (as mentioned also above for sensitivity analysis, statistical details should be added in methods and results)

Response: Thank you for the comment. Table 1 has been updated to include summary measures of the outcomes discussed in the submission. A second table has been included (Table 2) to further detail the lack of differences within the cohort with respect to participant sex, age, and maximum number of recorded bursts. We did not include further summary measures (beyond the range) for NNS outcome measures due to the longitudinal nature of the data. Differences between groups relating to NNS outcome measures is analyzed with the corresponding breakpoint analysis.
